# Review and outlook: from single nanoparticles to self-assembled monolayers and granular GMR sensors

**DOI:** 10.3762/bjnano.1.10

**Published:** 2010-11-22

**Authors:** Alexander Weddemann, Inga Ennen, Anna Regtmeier, Camelia Albon, Annalena Wolff, Katrin Eckstädt, Nadine Mill, Michael K-H Peter, Jochen Mattay, Carolin Plattner, Norbert Sewald, Andreas Hütten

**Affiliations:** 1 Department of Physics, Thin Films and Physics of Nanostructures, Bielefeld University, 33615 Bielefeld, Germany; 2 Institute of Solid State Physics, Vienna University of Technology, A-1040 Vienna, Austria; 3 Department of Chemistry, Organic Chemistry I, Bielefeld University, 33615 Bielefeld, Germany; 4 Department of Chemistry, Organic Chemistry III, Bielefeld University, 33615 Bielefeld, Germany

**Keywords:** bottom-up particle synthesis, dipolar particle coupling, granular giant magnetoresistance sensor, magnetic nanoparticles, self-assembly

## Abstract

This paper highlights recent advances in synthesis, self-assembly and sensing applications of monodisperse magnetic Co and Co-alloyed nanoparticles. A brief introduction to solution phase synthesis techniques as well as the magnetic properties and aspects of the self-assembly process of nanoparticles will be given with the emphasis placed on selected applications, before recent developments of particles in sensor devices are outlined. Here, the paper focuses on the fabrication of granular magnetoresistive sensors by the employment of particles themselves as sensing layers. The role of interparticle interactions is discussed.

## Introduction

Magnetic nanoparticles have been thoroughly studied during the last decades due to their many promising applications in chemical, physical and medical fields [1]. A common example is their employment in microfluidic devices: Due to their permanent magnetic moment, they can be controlled via external, inhomogeneous magnetic fields [2] and also be detected by magnetoresistive sensors [3] which allows for the magneto-based monitoring of magnetically labeled biomolecules.

The interaction between several particles is also of high practical relevance: Due to different types of coupling, magnetic nanoparticles assemble in superstructures. Various technological applications such as their employment in data storage devices, where every particle represents one bit of information [4], have been a strong driving force for the development of new methods for the well-defined deposition of superstructures on a substrate. In this regard, the different morphologies of nanoparticles have also become of interest as they offer additional degrees of freedom.

Within such assemblies, magnetic nanoparticles themselves may act as magnetoresistive sensor devices: Surrounded by a non-magnetic matrix, various spin-dependent transport phenomena have been observed [5–9]. Contrary to formerly used metallurgic preparation techniques, nanoparticle fabrication by bottom-up chemical syntheses offer significant advantages: The systematic adjustment of the self-organization process by, e.g., the employment of ligands with different alkyl chain lengths, allows for the independent variation of the particle-matrix volume fraction and the inter-particle distances between the magnetic granules and, therefore, enables a systematic study of granular resistive effects. These systems have promising applications of high technological relevance such as the realization of printable magnetoresistive sensor devices by the employment of colloidal magnetic spheres dispersed in a conductive paste.

However, the controlled preparation of highly ordered assemblies of magnetic nanoparticles requires a strong understanding of all steps involved and remains challenging due to the high degree of interdisciplinary influences. In this work, we give an overview of different preparation techniques, the resulting particles and the possibilities to control particle properties such magnetism of morphology by varying parameters in the synthesis process. The governing dynamics during the self-assembly process and within the static particle configuration are discussed, and we further analyze different properties of granular giant magnetoresistance sensors based on their spin-dependent transport properties.

## Review

### 1. Particle preparation

In principle, two different strategies for the synthesis of nanoparticles may be pursued. The top-down method starts from the bulk material which is decomposed by mechanical influences into decreasingly smaller fragments. The resulting objects have a mean diameter of about 100 nm and show a very wide size distribution. Therefore, such an approach is usually not suitable for the manufacturing of particles with a well-defined geometrical configuration.

The bottom-up method may be understood as an approach from the opposite direction: A small precursor, commonly an organometal compound or a salt, is decomposed by either thermal or optical excitation, which separates the metal atom from the organic residue, or by a reducing agent. Via the nucleation of numerous metal atoms, particles with a diameter of 1 to 50 nm and a narrow size distribution are formed. Due to the advantage of highly defined particle morphology, the bottom-up method is preferred in the works reported throughout this paper. However, a firm control of such properties for the design of particles tailored to specific applications requires a detailed understanding of different influences during the synthesis which are discussed in the following sections.

### 1.1 Thermolysis

**Figure 1:**
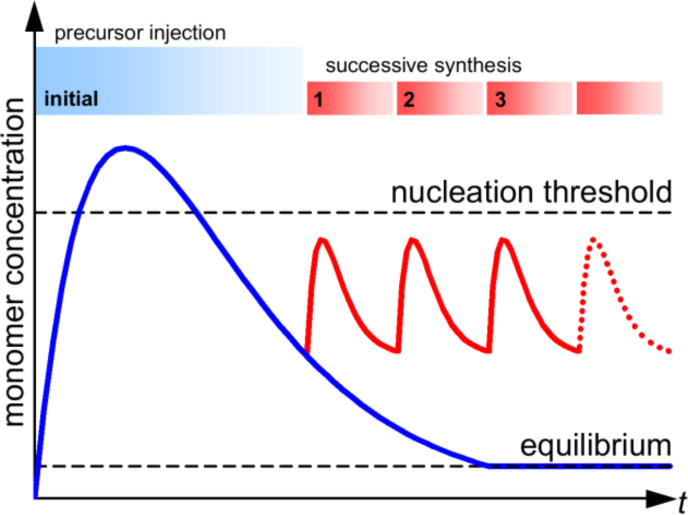
Schematic representation of the precursor concentration according to the LaMer model. The blue line represents the situation of a single injection; the particle size is limited by the precursor concentration. The red line shows the successive approach in order to increase the resulting particle size. During successive injection, the monomer concentration may not exceed the nucleation threshold.

A very commonly used method is thermolysis, which was originally introduced by Puntes et al. [10–11]. Tensides such as oleic acid, oleylamine, TOPO (tri-*n*-octylphosphine oxide), dendrimers or proteins are dissolved in airless conditions in an organic solvent and subsequently heated to reflux. By adding different organ metal compounds such as metal acetyl acetate [M(acac)_n_] or metal carbonyls, the formation of nucleation seeds is initiated. After formation, seeds absorb free metal atoms and continue to grow. The role of the tensides will be discussed below, however at this point, it is sufficient to know that they act as stabilizers for the particles; the resulting nanoobjects have a shell of the corresponding molecules. The particle growth dynamics can be explained in the frame of the LaMer model [12] which describes the growth process in two separate steps (Figure 1, blue line): above a critical concentration of free metal atoms, nucleation seeds are formed. Once the concentration drops below a critical threshold, the number of seeds remains constant and the existing seeds continue to grow.

From a thermodynamic point of view, nucleation seeds are formed once the nucleation energy barrier is exceeded. The free enthalpy Δ*G* is composed of surface contributions *G*_S_ and the bulk enthalpy *G*_V_:

[1]
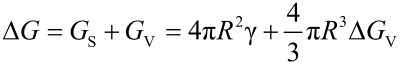


where *R* denotes the particle radius. The first summand describes the influence of the surface with γ the specific free surface energy. We always have γ > 0 and, thus, the nucleation process cannot be initiated due to surface effects. The second term refers to volume contributions with Δ*G*_V_ the free enthalpy difference between the solved monomer and the unit volume crystal. If Δ*G*_V_ > 0, solved monomers are energetically more favorable and, therefore, no nucleation seeds will be formed. For the synthesis of nanoparticles, it is, therefore, necessary to have Δ*G*_V_ < 0 such that *G*_S_ < |*G*_V_|. By introducing the degree of saturation *S*, Δ*G*_V_ may be rewritten as

[2]
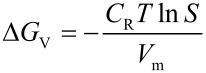


with *C*_R_ the Rydberg constant, *T* the absolute temperature and *V*_m_ the molar volume of the crystal. *S* reaches the value 1 for a completely saturated solution. At higher (supersaturated) concentrations, *S* > 1 and, consequently, also Δ*G*_V_ < 0. An analysis of the free enthalpy Δ*G* with respect to the particle radius *R* reveals that there is a maximum at

[3]
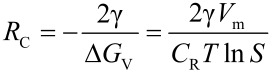


Below this critical radius, nucleation seeds can be formed, however, they immediately decay into smaller objects which are energetically more favorable. Therefore, the corresponding *R*-value *R*_c_ at the maximum of Δ*G* is the minimum size of a nucleation seed. The equations given above require the enthalpy difference Δ*G*_V_ and the specific surface energy γ to be constants. However, in the case of nanoparticles, this is no longer valid: Both values may strongly depend on the particle size and also different mechanisms of energy minimization such as rearrangement of the crystallographic phase may occur which are not included in Equation 1. Therefore, the critical size (Equation 3) is only an approximation.

Based on the LaMer model, the particle size can be controlled in different ways. Nucleation processes are initiated once the precursor concentration exceeds a critical concentration threshold. During the nucleation and in the subsequent seed growth, the concentration drops again below this boundary and no further seeds are formed. From this point onwards, the remaining free metal atoms contribute to the growth of the existing seeds. Therefore, the resulting particles are larger the less seeds have been formed during the nucleation events. Thus, particles with a large radius can be obtained by adjusting the precursor concentration to exceed the nucleation threshold by as little as possible which result in a small number of nucleation events. An alternative approach is indicated in Figure 1, red line, which is known as successive particle synthesis [13]. During the growth process, repeated injection of precursor concentration below the nucleation threshold results in a continuous growth without the formation of any new seeds. However, this method often leads to a broad size distribution.

In most synthesis processes, tensides form a basic requirement for particle stabilization: Due to their steric demand, they control the minimal distance between particles (see Section 2.1). If no tensides are present during the process, the synthesis will result in bulk material instead of nanoparticles. However, their interaction with the particle surface also proves key in the modification of particle properties: The interaction between a tenside and the particle surface can occur in many ways and are mainly based on dipole–dipole-, hydrogen bond- or van der Waals interactions. They do usually not show covalent characteristics.

**Figure 2:**
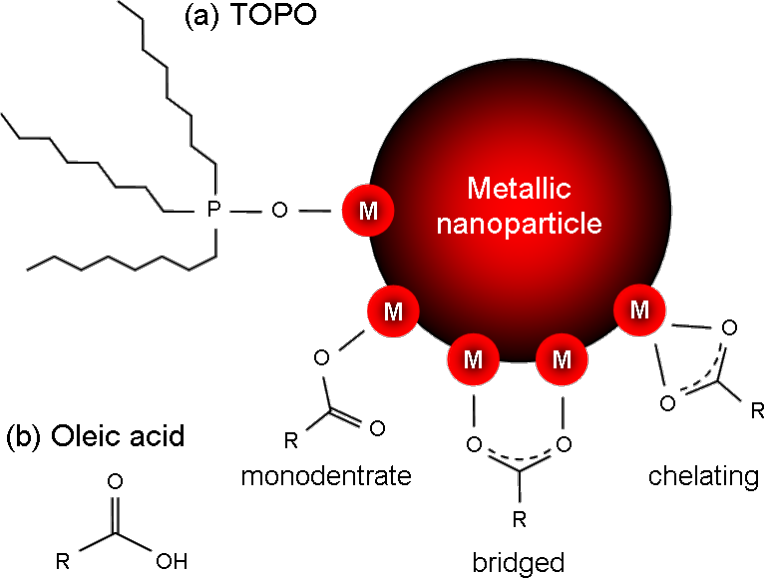
Interaction of different ligands with the surface of a metallic nanoparticle. There is only a single binding motif for TOPO (a) but three for ligands such as oleic acid (b).

Tensides can be characterized by their head groups via which they interact with metal atoms on the surface of the particles. We distinguish between tensides such as TOPO which has a phosphine oxide head group and can only bind in a single motif to the surface (Figure 2(a)) and tensides such as oleic acid where different binding motifs are possible (Figure 2(b)): In the monodentrate structure, only one oxygen atom binds to a metal atom, the second is not integrated. If both oxygen atoms are involved in the binding process, they form complexes with either two different metal atoms or a single one. These motifs are referred to as bridged and chelating, respectively (Figure 2(b)). Experimentally, the actual binding motif may be distinguished by IR spectroscopy due to a characteristically shifted carbonyl band [14]. Which motif is dominating for a specific tenside–particle pair depends on the properties of the metal surface and the structure of the head group of the absorbed tenside. In particular, lattice constants and crystallographic planes involved play an important role.

**Figure 3:**
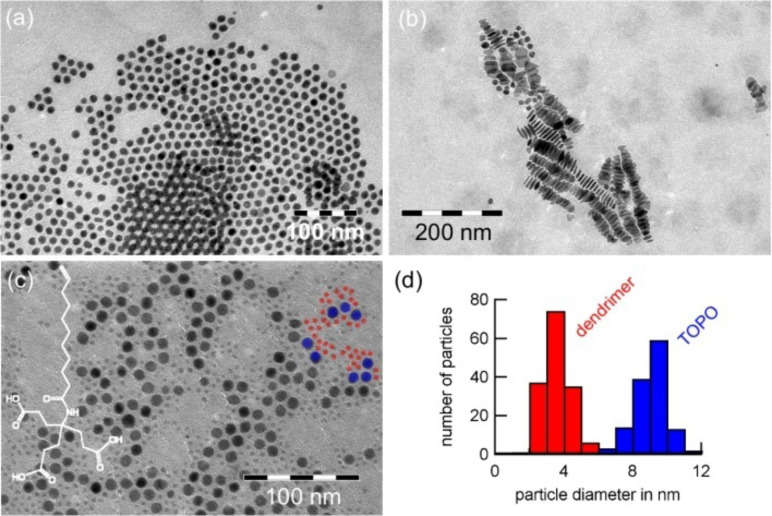
Transmission electron microscopy (TEM) images of Co particles of various sizes and morphologies synthesized under the influence of (a) TOPO, (b) oleic acid and oleylamine and (c) a dendrimer and TOPO which results in two distinct particle sizes (d).

The strength of the coupling between ligand and particle strongly affects the growth behavior of the metal cluster: The absorption of free metal atoms to the seed surface and, therefore, the continuation of growth is only possible at those areas where no complexes are present. A measure for the detachment of ligands is given by the dissociation constant *D*_e_. A small value of *D*_e_ corresponds to a hard to break bond between the metal surface and the ligand and, consequently, in reduced particle growth. The size of the dissociation constant may strongly vary, depending on the above mentioned binding affinities to different crystal planes. Crystals with a simple cubic symmetry result in an isotropic value which entails spherical particles (Figure 3(a)). However, if non-cubic crystal lattices are present, the dissociation constants may depend on the crystal plane and growth in specific directions is promoted [10,15–17].

Such effects are shown in Figure 3: The subplots present particles synthesized in *ortho*-dichlorobenzene employing dicobalt octacarbonyl as a precursor. As a ligand (a) TOPO, (b) a mixture of oleic acid and oleylamine and (c) a mixture of TOPO and a dendrimer of the first generation is present. The single binding motive of TOPO results in a constant dissociation along the particle surface and, thus, an isotropic growth. The multiple binding motives of the ligand mixture (b) lead to different binding affinities along different crystal planes. Therefore, the growth in specific directions is enhanced which can result in disk-shaped nanocrystals. In subplot (c), a bimodal particle distribution can be found. The two distinct sizes as shown in (d) result from different binding affinities of the tensides to the metal surface: Smaller particles are mainly stabilized by the dendrimer, larger ones by TOPO. The dendrimer has a very high dissociation constant which results in a strong binding to the metal atoms and, therefore, in a slow growth.

### 1.2 Alternative methods

#### 1.2.1 Micro emulsion and magnetotactic bacteria

**Figure 4:**
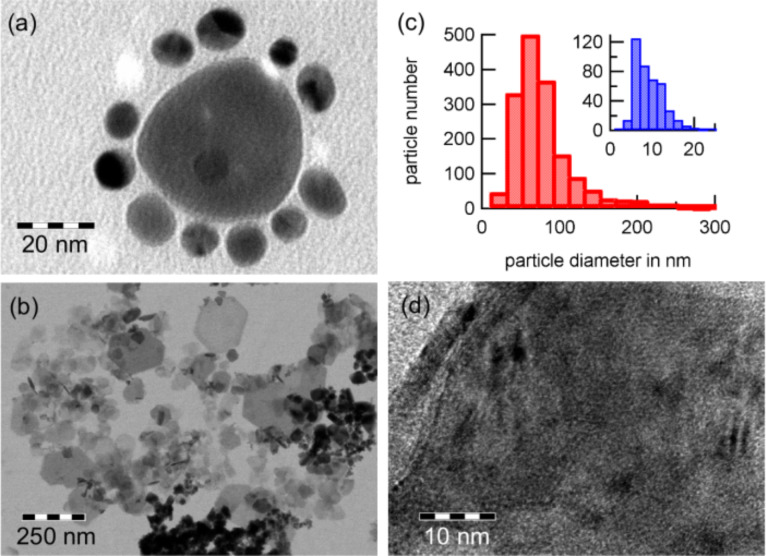
(a) Nanoparticles synthesized by micro emulsion approach and (b) by employment of the synthetic protein c25-mms6 after 15 days. The latter approach results in two different particle species of different sizes (c). Within a single particle, crystallites of different orientation can be found (d).

Another method for the synthesis of nanoparticles is the micro emulsion technique which is based on a thermally stabile, isotropic dispersion of two immiscible solvents, in which the micro domains of one or both solvents are stabilized by tensides on the boundary layer. Such behavior is well known from tensides in water which form micelles due to hydrophilic head groups and hydrophobic tails. Such micelles have a size of 1 to 50 nm depending on the tenside concentration [18]. The precursor is confined within these defined droplets which may, thus, act as nanoreactors in which particle growth is initiated. A typical result obtained by the use of an isopropanol/water emulsion and cetyltrimethylammonium bromide (CTAB) as a tenside is shown in Figure 4(a); the reducing agent is sodium borohydride.

While micro emulsion allows for much lower temperatures during the synthesis, stabilizing tensides usually need to be injected after the actual growth. Therefore, the additional control of the particle morphology by tensides is available. However, as shown by Tan et al. [19], it is still possible to synthesize nanoparticles of different shapes, materials or phases [20–21]. The major disadvantages of this technique are a broad distribution of size and morphology. Furthermore, much solvent is necessary for the synthesis which leads to a low efficiency in comparison to the thermolysis.

**Figure 5:**
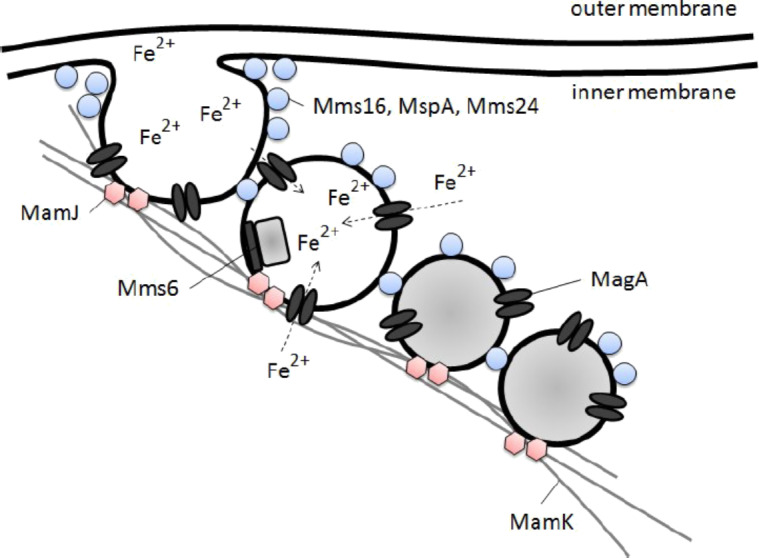
Hypothesized particle formation during the biomineralization process in magnetotactic bacteria (in analogy to [22]).

A very similar mechanism can be found with magnetotactic bacteria which produce ferrite nanoparticles under mild conditions as part of their metabolism. The biomineralization process within such bacteria is not yet well understood. Recent studies indicate specific genes and proteins play a major role [22]. As shown in Figure 5, the growth dynamic is believed to be a multistep process [22–23]:

1. *Invagination of cytoplasmic membrane*: The cytoplasmic membrane invaginates for vesicle formation. These vesicles later serve as precursors of the nanoparticle membrane. It is believed that a 16 kDa protein Mms16 (small GT-Pase) assists with the vesicle formation. A second protein Mms 24 (24 kDa) may also be required [24].
2. *Accumulation of ferrous irons*: External iron ions are transported into the vesicle. Ferric iron Fe^3+^ appears to be reduced on the cell surface and transported into the vesicle as ferrous iron Fe^2+^. This conversion is required so the iron ions can pass the cytoplasmic membrane, a detailed description can be found in [25]. A protein magA appears to be involved in this transport process. The oxidation level within the vesicles is controlled by an oxidation–reduction system.
3. *Nucleation*: Several proteins are believed to regulate the morphology. Mms5, Mms6, Mms7 and Mm13 are tightly bound to the magnetic nanoparticle. All these proteins are amphiphilic. Their *N*-terminal is hydrophilic while their *C*-terminal is hydrophilic. The hydrophilic *C*-terminal of Mms6 is believed to be the iron binding site [26].

Recent studies within our group showed that nanoparticles can be synthesized in vitro by the use of a shorter synthetic version of the protein Mms6 called c25-mms6. This polypeptide consists of 25 amino acids from the *C*-terminal region of Mms6. In this study, cobalt ferrite nanoparticles not known to occur in magnetotactic bacteria were synthesized. Cobalt and iron salts were added to the c25-mms6 mixture and incubated at 4 °C. The mixture was stirred under argon flux until it reached room temperature and then left for 15 to 28 days to allow for crystal growth. The nanoparticles obtained can be divided into Co_2_FeO_4_ and CoFe_2_O_4_ particles, Figure 4(b,c), which consist of small phase separated crystallites, Figure 4(d). The majority of larger particles is hexagonally or truncated hexagonally shaped and constitute the Co rich phase. A control experiment without c25-mms6 showed that the nucleation is not triggered by the protein but that it regulates shape and morphology and, therefore, the physical properties of the nanoparticles.

#### 1.2.2 Bimetallic nanoparticles

**Figure 6:**
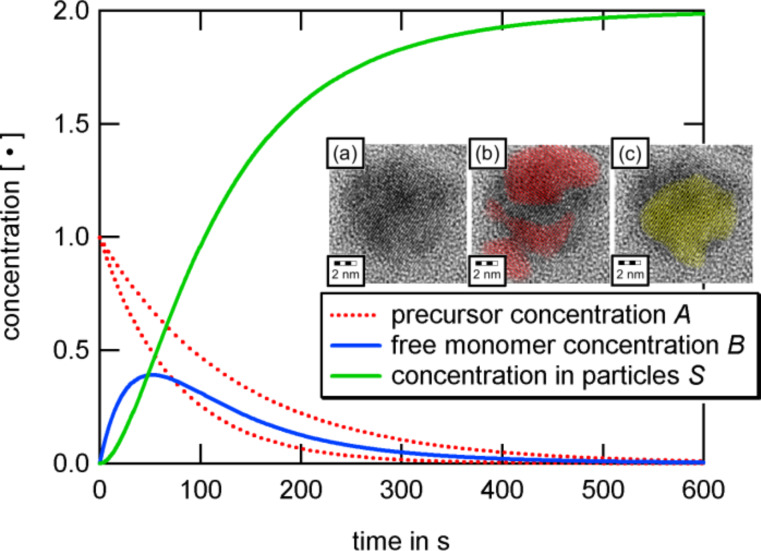
Dynamic evolution of different concentrations for the decay rates *k*_1_ = 0.00753 s^−1^ and *k*_2_ = 0.0136 s^−1^ and *k*_3_ = 0.03 s^−1^ for the respective precursors A_1_ and A_2_ and the free monomer B. The insets show a high resolution TEM image of a Fe_0.47_Co_0.53_ nanoparticle (a) which has been synthesized via such an approach. The particle is oriented in bcc (100) direction. The center of the particle consists of a Co enriched alloy (Fe_0.44_Co_0.56_, marked by a yellow sublattice (c)) while the surface shows a higher Fe content (Fe_0.63_Co_0.37_, red lattice (b)).

Bimetallic nanoparticles [27–28] form an important area in the field of nanoparticles based on their interesting properties which provide various advantages in comparison to monometallic nanocrystals. An example can be found with CoFe particles which have a strongly increased magnetic moment per atom in comparison to pure Co particles [29]. Bimetallic particles can be classified into 5 groups [30]:

1. *Stoichiometrical compounds* with well defined crystal structures. Examples are CdSe semiconductor particles or magnetic FePt particles [31].
2. *Undefined mixtures*. Two compounds are completely miscible. This situation occurs if the bulk metals have similar structures with a mismatch of below 10%. SiGe [32] and AuAg [33] are systems of this type.
3. *Undefined structure with a concentration gradient*. The requirements are similar to the second class but the component distribution is controlled kinetically. CoFe is a well known example [30] (see Figure 6).
4. *Core shell particle*. Based on two immiscible materials, one compound in the center (core-phase) is coated by the second (shell phase) [34].
5. *All other two phase systems* which are not in class 4. Similar requirements as in class 4 need to be met [35].

Thermodynamic and kinetic properties influence the type of particle which results from the synthesis. Depending on the miscibility of the two compounds, either a single phase system (1–3) for high miscible or a two phase particle (4,5) in case of immiscible components is obtained. A first estimation on the miscibility can be concluded from the phase diagrams of the bulk materials.

In order to illustrate the growth dynamics and material distribution along the particle volume, we consider two miscible compounds, A_1_ and A_2_, such as iron and cobalt carbonyl. The result should fall into the classes 1 to 3. Precursors decay at different decay rates *k*_i_ which entails a high concentration of the less stable precursor in the particle center and a bimetallic particle of class 3 [13]. According to the LaMer model, precursors decay and free monomers B are formed. If concentration and initial concentration are denoted by [•] and [•]_0_, respectively, the equations of evolution are given by

[4]
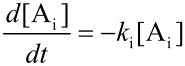


[5]
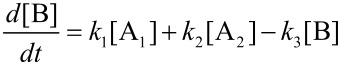


with the solutions (Figure 6)


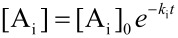



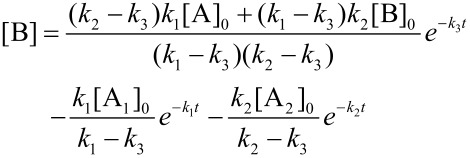


The absolute concentration of the material absorbed by nucleation seeds is [S] = [A_1_]_0_ + [A_2_]_0_ − [B] − [A_1_] − [A_2_] and, therefore, the particle growth rate is given by *v*(*t*) = *d*[S]/*dt*. Further, the ratio *x* = A_1_/A_2_ of material absorbed at time *t*


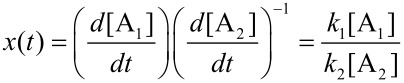


with the relative compound ratios


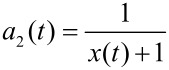


and


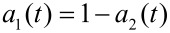


allows for determination of the inner distribution of the two compounds via integration of the individual growth rates *a*_i_*v*, with respect to time.

In a different approach, monometallic particles are synthesized in a first step and subsequently coated by a second metallic compound which can be realized, e.g., with the successive method described in the LaMer model, by changing the precursor solution during the injections. This approach results in core–shell nanoparticles [36]. Also, it is possible to protect the core of a magnetic particle by different materials, e.g., in order to stabilize the material against oxidation or to allow for the employment of toxic materials in biomedical applications [37–38].

### 1.3 Magnetic properties

In the subsequent sections, we will mainly focus on magnetic properties of assemblies of nanoparticles. As the components of such assemblies, it is necessary to understand the properties of individual nanoparticles themselves. In comparison to macroscopic objects, nanoparticles have a very high ‘surface to volume’ ratio and are on the size scale where quantum mechanical effects are increasingly of more importance. Therefore, the magnetization of nanoparticles is dominated by finite size and surface effects [39–40].

The magnetic structure of macroscopic magnetic materials is divided into magnetic domains. Along these domains, magnetic moments have a parallel alignment, different domains are separated by domain walls. In comparison to a homogeneously magnetized object, the formation of domains decreases the magnetostatic energy of the system proportional to the sample volume. However, a certain amount of energy is required for the creation of the domain wall, which is proportional to the domain interface. With the reduction of the sample size, interface effects gain importance until below a critical diameter *d*_c_, the formation of domains is energetically less favorable. For spherical particles, this critical diameter *d*_c_ depends on various material properties such as the exchange constant *A*, the effective anisotropy constant *K*_eff_ and the saturation magnetization *M*_S_, and is given by


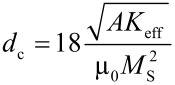


where µ_0_ is the vacuum permeability.

In this work, we focus on particles of sizes between 5 to 20 nm; the single domain limits of cobalt and iron nanocrystals are on this size scale. The crystalline microstructure introduces energetically favorable easy axes and directions of high energy, hard axes. The magnetization of a free particle aligns with one of the easy axes. In order to switch the magnetization into a different state, a certain energy barrier needs to be overcome. If this energy originates from thermal energy, particles are called superparamagnetic. There are no longer stable magnetization configurations but the magnetic moment permanently switches between different orientations. For uniaxial crystal anisotropy, the superparamagnetic size limit needs to meet

[6]
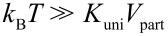


where *k*_B_ is the Boltzmann constant, *T* the absolute temperature, *K*_uni_ the first anisotropy constant and *V*_part_ the particle volume. In particular, we can directly derive the superparamagnetic radius *R*_spm_

**Figure 7:**
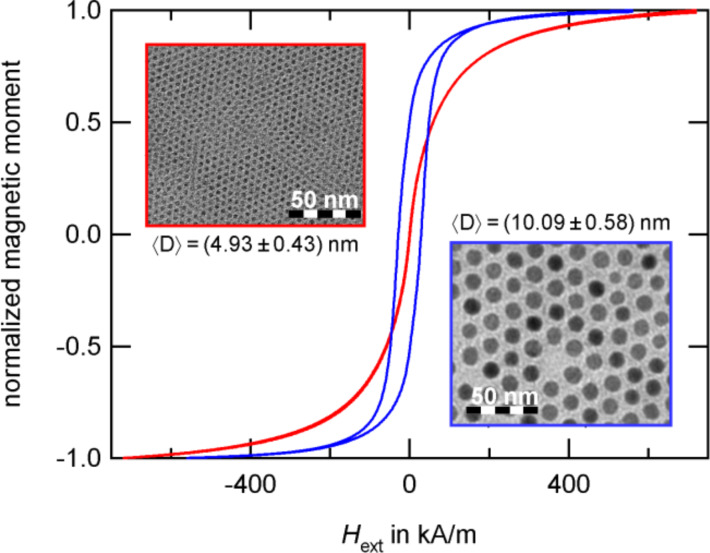
Co particles with a diameter of 4.9 and 10 nm measured at room temperature shortly after the preparation of particles. With the superparamagnetic limit at 7.8 nm [13], the larger particle species (blue) shows a hysteresis while the small particles (red) are superparamagnetic at room temperature.

[7]
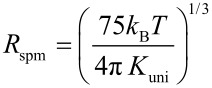


below which superparamagnetic behavior can be found. For spherical hcp Co particles, this is expected at a diameter of 7.8 nm [13]. Superparamagnetic particles show no hysteresis; their magnetization response to an external magnetic field resembles the Langevin behavior of paramagnetic materials but with the high susceptibility and magnetization values of the ferromagnetic materials they are composed of, compare Figure 7.

With even smaller particles, surface effects become dominant and a fully quantum mechanical treatment is necessary for their description. For example, 60% of all spins of the 1.6 nm fcc Co particles analyzed by Batlle et al. are surface spins [39]. Particles in this size scale lie outwith the scope of this work. We will only consider particles, where the semi-classical treatment is a good approximation.

### 2. Self-assembled particle structures

**Figure 8:**
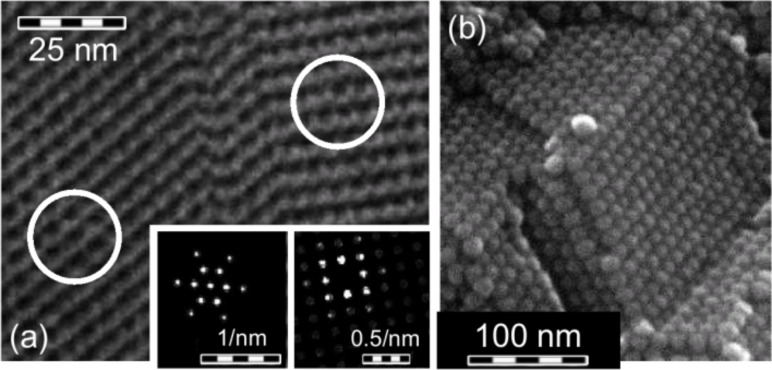
Self-assembled FeCo nanoparticles with different dimensions: (a) The 2D-monolayer of 4.6 nm sized spherical FeCo nanocrystals shows a phase transition from a hexagonal to a cubic lattice symmetry. The FFT patterns are taken from the marked areas. (b) SEM image of a millimetre sized 3D supercrystal composed of FeCo particles with a diameter of 15 nm. The crystal has been broken to show the high degree of order inside. (Figure (b): Reprinted by permission from Macmillan Publishers Ltd: Nature Materials, ref. [48], copyright 2005, http://www.nature.com/naturematerials)

The ability of nanoparticles to self-assemble on a substrate has opened the way to many applications such as sputtering masks, magnetic data storage media or sensor devices [41–44]. This interesting phenomena can result in highly ordered regions ranging from monolayers of hexagonally or cubically ordered arrays with sizes between a few square nanometers up to the square micron scale [13,45–47] and to three dimensional superlattices of several cubic millimeters [48–49] as shown in Figure 8. For many applications, a high degree of order on a large scale is essential; we will see an example for this later on in Section 4. In order to obtain such highly symmetric particle patterns, a narrow particle size distribution is essential; the standard deviation should not exceed 10% of the mean value [50–52]. As already mentioned in the preliminary section, bottom-up synthesis methods are well suited for these requirements. In addition, particle size distributions can be further refined via sedimentation or by magnetic separation subsequent to chemical synthesis [15].

### 2.1 Driving forces to self-assemblies

The organization process is driven by a superposition of interparticle interactions and external forces [47–53]. Interparticle forces act on the nanocrystals in the liquid phase of a particle solution as well as during the assembly process on a substrate. Different forces may have major impact on the resulting assemblies: An attractive potential is given by the van der Waals interaction which is caused by induced electric dipoles and acts along the connection line between them. For two interacting solid spheres Hamaker derived the expression

**Figure 9:**
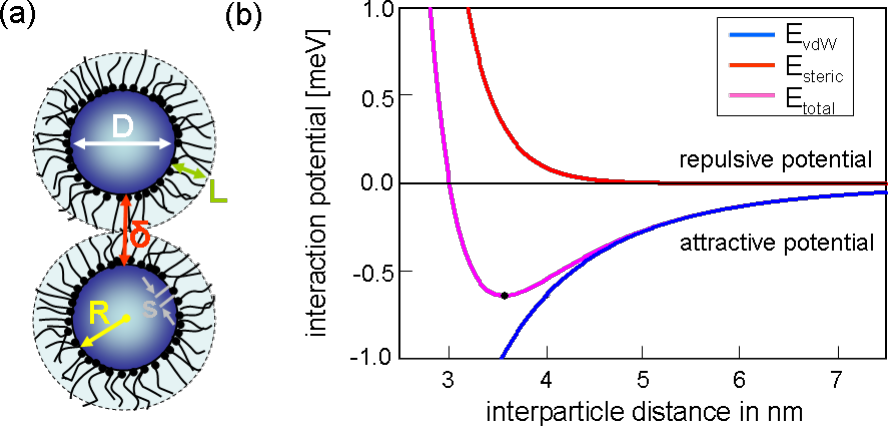
(a) Scheme of two particles with a metallic core of radius *R* surrounded by a ligand shell of the thickness *L*, *s* denotes the required distance of two neighboring ligand head groups on the surface of the particle cores and δ the surface distance between the metallic cores. (b) Total interaction potential calculated from the contributions of the steric repulsion (red) and the attractive van der Waals potential (blue) for two Co nanoparticles of a diameter of 3.3 nm stabilized with oleic acid (*L* = 1.17 nm, *s* = 0.51 nm).

[8]
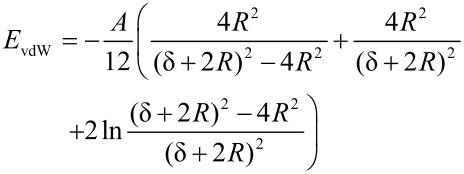


for the interaction potential [50–52] with *A* the Hamaker constant, and *R* and δ the particle radius and the interparticle distance, respectively (compare Figure 9 (a)).

Repulsive force contributions originate either from electric Coulomb forces or steric repulsion, depending on the nature of the particle stabilization. For instance, spherical particles which are surrounded by a dense ligand shell with non-polar end groups result in a short ranged repulsive potential that can be calculated by the equation of de Gennes [54]

[9]
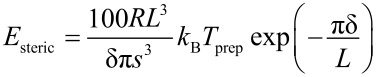


where *L* is the thickness of the ligand shell, *s* the distance of two neighboring ligand headgroups on the surface of the particle core, *T*_prep_ the absolute temperature during preparation and *k*_B_ the Boltzmann constant. This potential strongly depends on the properties of the employed ligand. Therefore, ligands do not only play a key role for the geometrical properties of individual particles but also for the organization of ensembles in superstructures. Figure 9(b) shows the different potential contributions calculated according to Equation 8 and Equation 9, if the parameters of oleic acid stabilized Co nanoparticles with a diameter of 3.3 nm are assumed (*T* = 400 K, *L* = 1.17 nm, *s* = 0.51 nm). The superposition of both potentials results in a total potential with a global minimum. In the example, the particles will assemble at a distance of about δ = 3.6 nm.

**Figure 10:**
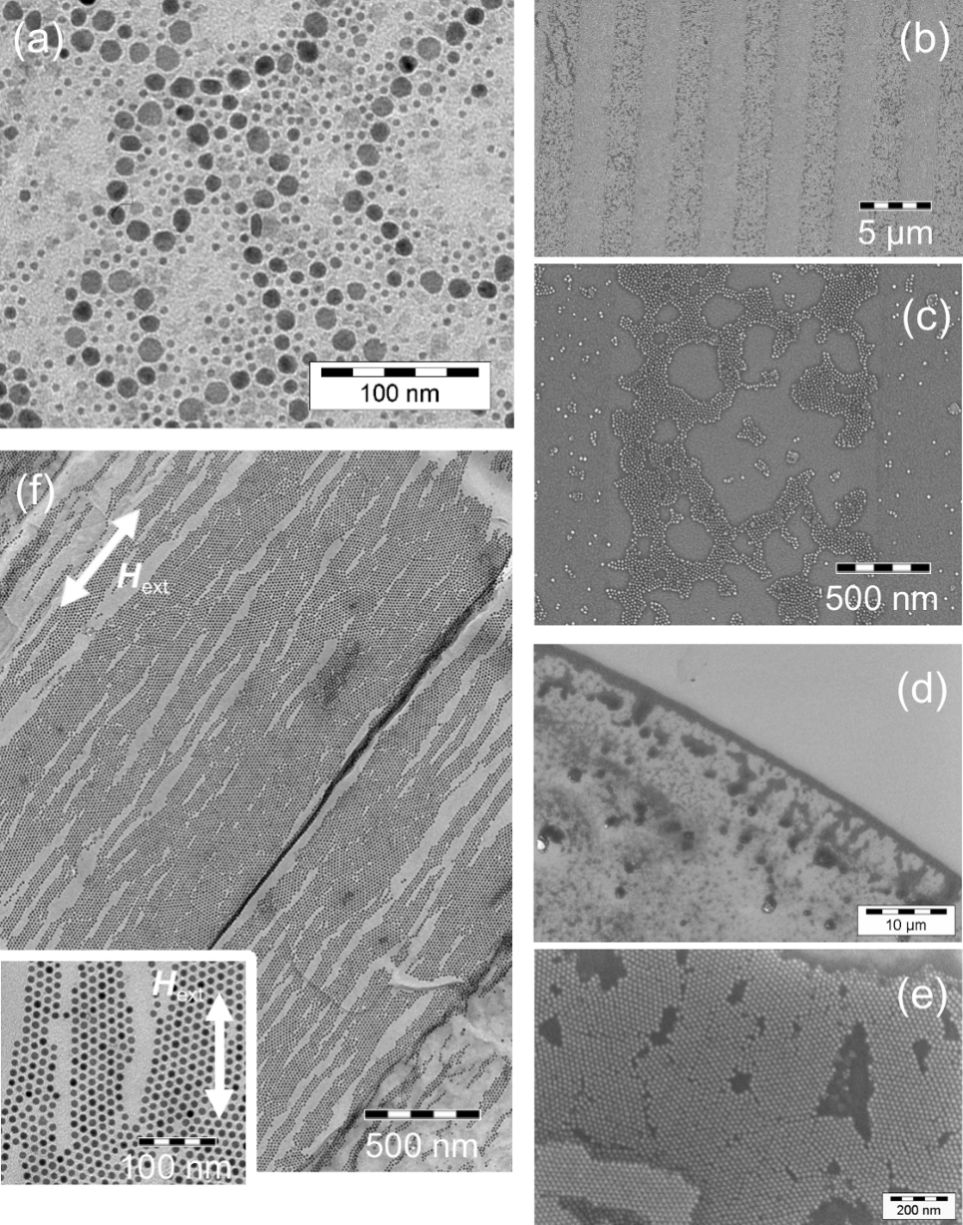
Images of self-assembled spherical Co nanoparticles: (a) TEM image of a bimodal distribution; large particles with a diameter of about 12 nm are ferromagnetic and assemble in ring-shaped superstructures. (b) Influence of the surface properties on the self-assembly; Co particles are predominately arranged along areas which were previously covered with photo resist and (c) detailed image of the particle ordering. (d) SEM images of assemblies under the combined influence of hydrodynamic and capillary forces; the edge of the drop mainly consists of a particle monolayer. (e) Detailed image of the hexagonal network formed by the particles within the monolayer. (f) Particles deposited under the influence of an external magnetic field. The nanocrystals are organized in lines oriented parallel to the direction of the applied field. The inset shows a detailed image of the distorted hexagonal order.

For magnetic particles with sizes above the superparamagnetic limit (Equation 7), dipole–dipole interactions between adjacent particles can play a major role during self-assembly. Such ferromagnetic particles mutually align their magnetic dipole moments which entails an attractive coupling and may result in different geometrical patterns such as particle chains or rings [55–56]. An example of a dipole interaction dominated arrangement is shown in Figure 10(a): Co particles with a bimodal size distribution show varying behavior depending on their size. The hcp Co particles of a diameter of 12 nm are above the superparamagnetic limit and self-assemble in chain superstructures while the smaller particles are superparamagnetic and favour a hexagonal ordering.

Recent developments on the directed assembly of nanoparticles under external influences have attracted much interest. Such constraints may arise during the self-assembly process on a substrate or by exerting the particle solution to external electromagnetic fields. Since this topic is not quite within the focus of this work, we will only show a few possibilities.

Convective particle flux may be induced by a hydrodynamic velocity field within the solvent on top of a substrate. The effect is shown in Figure 10(d): a droplet of particle solution with heptane as a solvent was placed on a SiO_2_ surface. The spreading of the droplet results in a force onto the particles which entails the assembly close to air–liquid boundary. This allows for a controlled positioning of the particle monolayer within a specified target region (on top of magnetoresistive sensors, between contacts for measurements of electrical transport properties etc.) if the drop parameters such as volume–diameter relation for a specific solvent-substrate combination are known. Further, capillary forces improve the ordering of the particle monolayer along the edge (Figure 10(e)). This attractive force is caused by the Laplace pressure which arises when a curved meniscus is formed around two adjacent particles during the evaporation of the solvent. Due to the linear dependence of the capillary force on the particle diameter, the action is stronger the larger the particles. Therefore, although suspended in the same solvent, smaller particles show a lower degree of order [49].

Additionally, friction and shear forces can arise between the particles on the one hand and between particles and substrate on the other hand [57–58]. In the latter case, the forces strongly depend on the surface properties such as structure and roughness. Thus, the choice of substrate is another crucial factor for the preparation of homogeneously ordered superlattices on large scales. The influence of different surface conditions is shown in Figure 10(b): A photo resist mask of 2.5 µm wide strips created on top of a Ta layer by optical lithography was employed. After removal of the mask, the substrate was dipped into a particle suspension. Along the formerly resist covered area, a higher particle density can be observed. This effect can be attributed to a strengthened adhesion within the strips due to a modified surface roughness and energy.

In order to obtain a magnetically structured sample, a suspension of ferromagnetic particles can be placed on the substrate in the presence of an external magnetic field. For manufacturing of particle layers, a homogeneous magnetic field needs to be employed; inhomogeneous fields result in the accumulation of nanoparticles along the area where high field gradients can be found [59]. An example of ferromagnetic Co nanocrystals arranged under the influence of a homogeneous magnetic field of 120 kA/m parallel to the substrate plane is shown in Figure 10(f): Particles arrange along lines parallel to the external field which is in contrast to free self-assembly (Figure 10(a)). The magnetic orientation within the nanocrystals is dominated by the external field which results in a distortion of the hexagonal ordering due to repulsive forces between adjacent lines of particles perpendicular to the field direction.

### 2.2 Influence of the particle geometry

**Figure 11:**
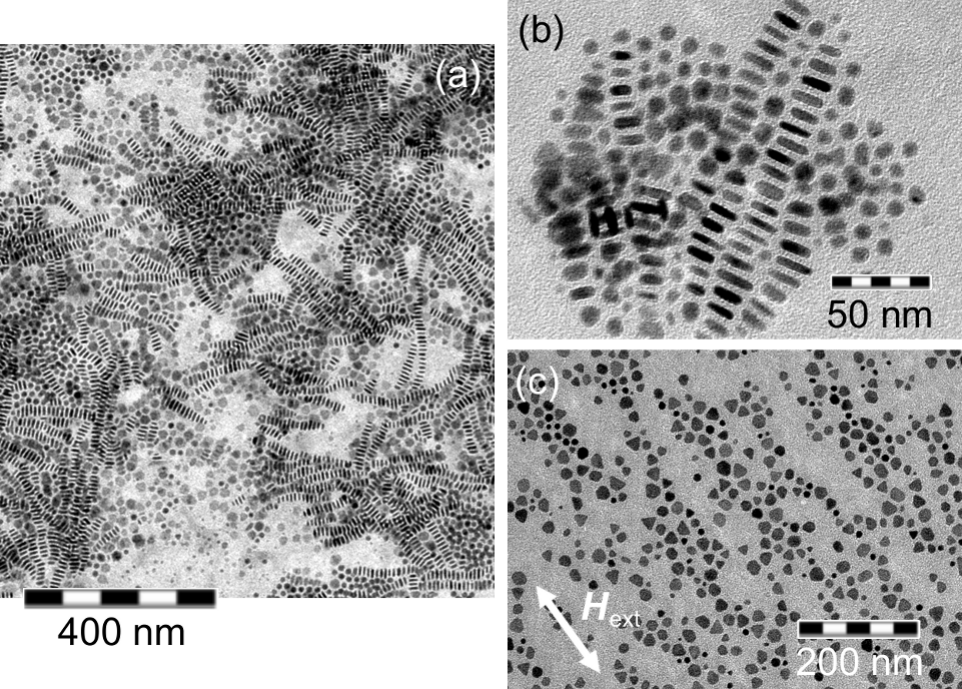
TEM images of self-assembled Co particles with different morphologies. Nanodisks exhibit a typical thickness to diameter ratio of 1:3 and organize mainly in rows of standing disks. Three-dimensional superstructures can be observed when two rows of disks cross each other (a). Assemblies of Co nanodisks deposited on a TEM grid (b) without and (c) under the influence of an external magnetic field of *H*_ext_ = 160 kA/m applied parallel to the substrate plane.

As already discussed in the preliminary section, the particle morphology can be controlled by appropriate ligands. In contrast to spheres, nanocrystals with the shape of (truncated) triangles, facetted particles or hexangular disks have additional rotational degrees of freedom. Figure 11(a) shows a sample of Co particles with a broad distribution of different shapes. In particular, the disk-shaped objects show interesting behavior: They are mainly arranged in long rows of up to 40 disks, stacked face-to-face and standing on their edges. Within the two-dimensional TEM image, disks resemble the shape of rods. However, on tilting the sample, they may easily be identified as nanodisks [10,15]. A more detailed analysis of the rows reveals a size gradient along the superstructures; disks of larger radius are placed further towards the center [51]. Individual rows of disks propagate in a random direction and adherent rows tend to align with each other in areas of high concentration [10,60].

The arrangement of disks is not yet completely understood. The minimization and size dependence of the van der Waals contribution are supposed to be the main driving forces for the spatial arrangement [15,51] and the size distribution along the chains [51]. Bao et al. explained the formation of disk rows by a hydrophobic interaction between the ligand tails, thus, minimizing exposure to air by maximizing the contact between the ligand tails [60]. Due to the magnetic nature of the Co disks, a magnetic origin for the formation of rows is also under discussion. Under the influence of strong shape anisotropy, the magnetization direction is confined within the disk plane which was believed to entail an antiferromagnetic configuration in order to minimize the magnetic stray field along a particle row [15,46]. However, in 2006, Gao et al. [61] performed electron holography experiments on magnetic Co disks which reveal a spiral-like arrangement of individual moment vectors around the row propagation axis.

Similar to the situation of spherical magnetic particles, the orientation of such disks can be controlled by the application of an external field during the deposition [15]. Figure 11(b) shows a typical arrangement of nanodisks if no external field was applied during the self-assembly. By applying an in-plane magnetic field of 160 kA/m, the configuration shown in Figure 11(c) is obtained. This allows for several conclusions: a) The disk plane coincides with the magnetically easy plane of the nanocrystals and b) the driving forces responsible for the self-organization process may be overcome by the magnetic interactions induced by the homogeneous external field.

### 3. Magnetically interacting nanoparticles

As already demonstrated in the preliminary section, different types of interactions entail self-organization processes of magnetic nanoparticles in chains or monolayers depending on the geometry of magnetic objects and external constraints. However, once the geometric configuration has reached an equilibrium state, remagnetization processes along the assembly become the dominating dynamics. Since small magnetic objects do not have an inner magnetic substructure but the magnetization is homogeneously distributed along the volume, the stray field at a point **r** of a magnetic nanoparticle with magnetic moment **m** situated in the origin is given by the dipolar expression [62]

**Figure 12:**
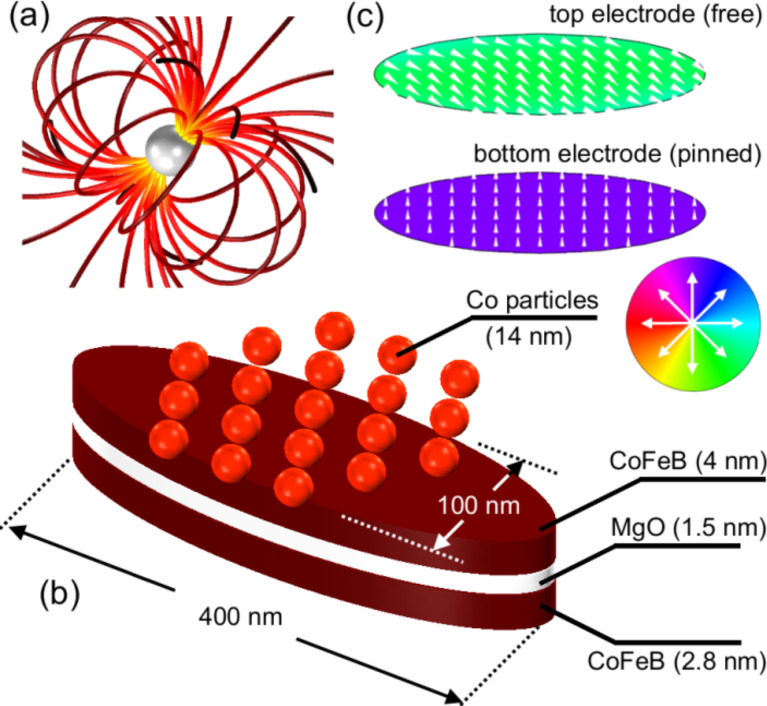
Schematic representation of the tunnel magnetoresistance (TMR) sensor setup for the detection of multiple Co nanoparticles of 14 nm diameter: (a) Stray field of a homogeneously magnetized particle, (b) TMR-sensor with particles on top, (c) magnetic equilibrium state of the ferromagnetic electrodes.

[10]
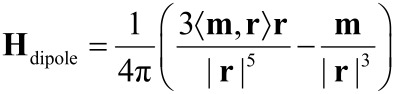


with ‹ • , • › the Euclidean inner product. A schematic representation is shown in Figure 12(a). Adjacent particles influence each other via their dipolar coupling. Strong interactions can be found in such assemblies which even cause agglomerations of superparamagnetic components to show hysteretic behavior.

### 3.1 Direct observation of dipolar coupling

In order to analyze the magnetic properties of assemblies of magnetic nanoparticles, tunneling magnetoresistive (TMR) sensors are employed. The schematic configuration of a TMR sensor is shown in Figure 12(b): Two thin ferromagnetic films are separated by an insulating barrier [63]. If the TMR sensor is positioned in an external magnetic field and a bias voltage is applied across the stack, then a quantum mechanical tunneling current flows across the insulator barrier. The resistance of the TMR sensor depends on the relative orientation of the magnetization within the two ferromagnetic layers [64]. A perturbation field introduced by a single magnetic particle or by an assembled monolayer of them entails a variation of the magnetization distributions in both electrodes which leads to a change of the measured resistance. Depending on the resistance change, different conclusions on the configuration on top of the sensor surface may be drawn. In order to enhance the effect and to allow for a wide range of applications, the top electrode is usually chosen magnetically soft to be easily influenced by magnetic field variations to be detected, while the bottom (reference) layer is magnetically hard and, ideally, cannot be switched by external perturbations.

The experimental setup employed here consists of two CoFeB layers that are separated by an MgO barrier. The geometric and magnetic configuration of the sensor is chosen to allow for a precise measurement of single magnetic beads and nanoscale objects: We employ elliptically shaped sensors with longitudinal and lateral dimensions of 400 and 100 nm, respectively. The magnetic configuration within the top electrode is free to rotate while magnetization of the lower CoFeB layer is fixed by an artificial antiferromagnet. From micromagnetic simulations, we can conclude the equilibrium magnetic configuration of the free sensing layer: Without any external influences, the magnetization would align parallel to the long ellipse axis. However, due to the stray field of the pinned bottom electrode the magnetization orientation is tilted towards an antiparallel configuration with respect to the reference layer (Figure 12(c)). For a more detailed description on the sensor configuration, fabrication and properties, see [65–66]. The interplay between geometrical shape anisotropy and stray field coupling of the layers entails a resistance change which is linearly connected to the strength of an external magnetic field in a field range of ±40 kA/m. Thus, such sensors are well suited for the detection of multiple particles. Due to the linearity in the response, we expect a signal proportional to the number of particles deposited on top of the sensor surface.

For the experimental realization, 14 nm Co particles were deposited on top of the sensor via a dropping procedure which results in random particle distributions along the surface. In order to compare different sensors, we analyze the relative change

**Figure 13:**
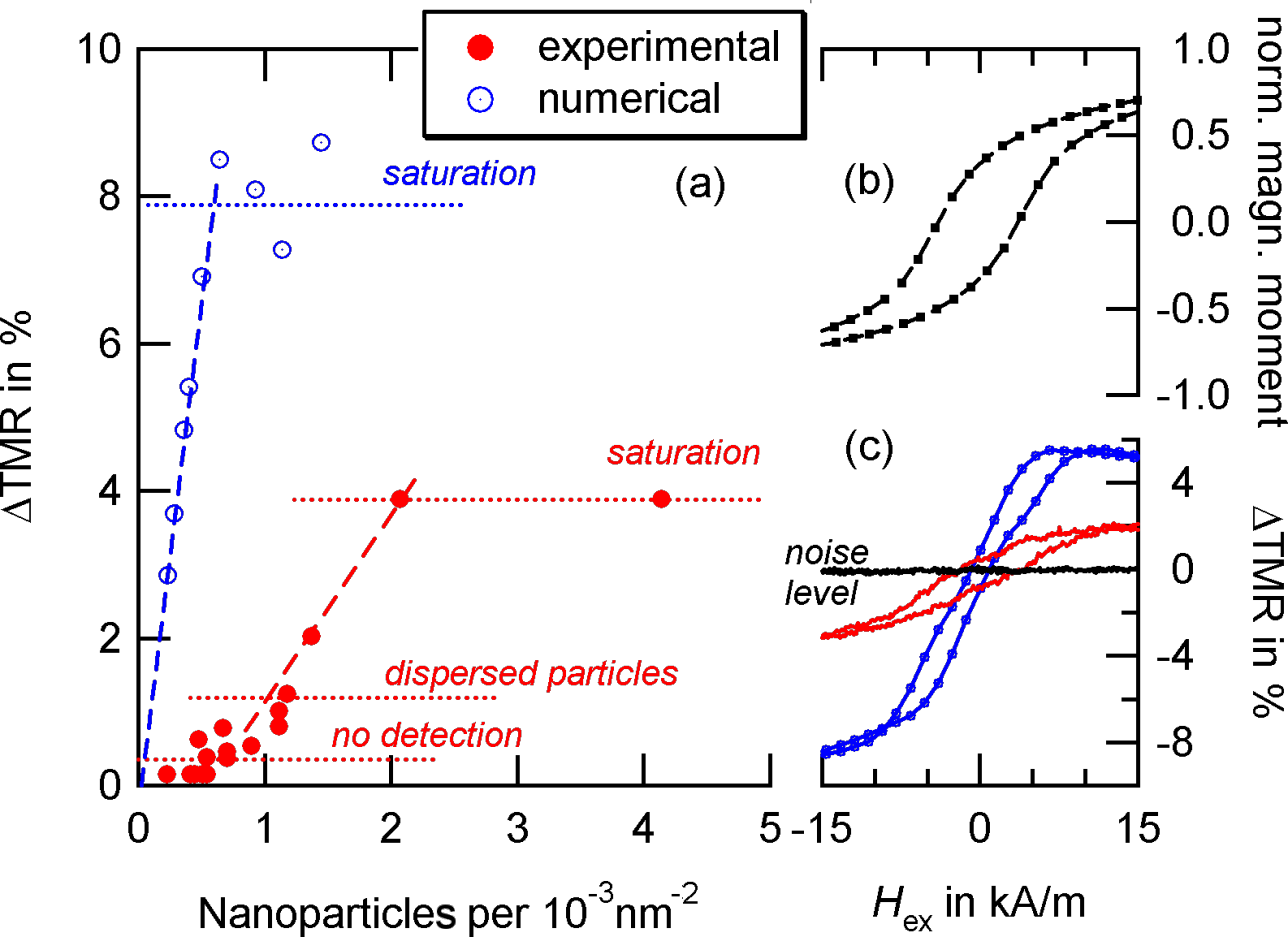
Properties of the magnetoresistive sensors. (a) Comparison between experimental and numerical data. The magnetic properties of the 14 nm Co particles obtained by AGM measurements (b). The coercive field *H*_C_ = 3.76 kA/m entails a hysteretic ΔTMR-signal (c) due to the detection of nanoparticles that interact by dipolar coupling.

[11]
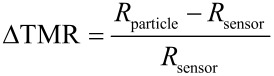


with the respective resistances *R*_particle_ and *R*_sensor_ for the situations with and without particles on top. The experimental measurements are shown in Figure 13(a) (red markers). For a very small number of particles, the measured signal is below the electric noise ratio of the device and no detection is possible. Once a critical detection threshold is exceeded, a linear increase, corresponding to the degree of coverage, can be reported, as expected. With the dipolar coupling strength decaying by 1/*r*³, the distance between nanoparticles proves crucial for the observed behavior. In particular, if particles are freely dispersed on the top of the sensor and sufficiently far apart from each other, the induced magnetic moment resembles the intrinsic anisotropy of the nanoparticle [67]. The detected signal shows no hysteresis which reveals the superparamagnetic nature of the nanoparticles. Moreover, the intensity of the detected signal increases linearly with the number of particles situated on the sensor surface; similar results have been reported by Wang and Li [68]. With decreasing particle distances, the significance of dipolar coupling increases. A manifestation of this type of interaction is the induced hysteresis in the detection signal. The coercive field of the ΔTMR-hysteresis loop coincides with the coercive field measured by an alternating gradient magnetometer (AGM). Once a second critical value is exceeded, no further increase can be reported; the signal remains constant.

In order to understand the experimental observations, the findings are compared to numerical simulations: Particles are assumed to be organized along a hexagonal grid on top of the sensor as shown in Figure 12(b); the surface concentration is modified via the adjustment of the lattice parameter. The magnetodynamics of *N* homogeneously magnetized particles are governed by a set of ordinary differential equations [69–70]

[12]
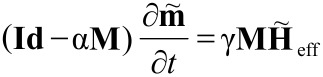


where **Id** is the 3*N* × 3*N* identity matrix, γ the gyromagnetic ratio, α the empirical damping coefficient and further the block diagonal matrix **M**


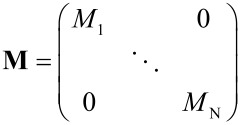


where 
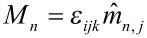
, *n* = 1, … , *N*, and the vectors


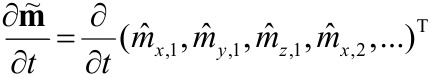



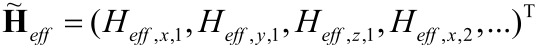


.

For the effective magnetic field, we restrict our analysis to pure dipolar coupling. Therefore, each magnetic moment evolves under the influence of the superposed stray fields of adjacent particles. Due to a 1/*r*^3^-decay, it is sufficient to neglect the interaction of particles with a distance more than five times the average particle radius [71]. By integrating Equation 12 and employing the solution of the equilibrium configuration for micromagnetic simulations of the free sensing layer, the data shown in Figure 13(a) (blue markers) are obtained.

In qualitative agreement to the experimental observation, a linear increase for low surface coverage can be found while for high concentrations the signal becomes stationary (Figure 13(a)). Further, the ΔTMR-response shows a hysteretic behavior (Figure 13(c), blue line). Quantitatively, the numerical data predict a sensor response which is about double the value at half the particle concentration in comparison to the experimental findings. Also, the hysteretic signal observed in the experiments is about double the theoretical value. These particular deviations may be attributed to the highly idealized particle distribution on top of the sensor: According to the preliminary section, (ferro-)magnetic particles form self-assembled structures and agglomerations in the liquid phase (Figure 10(a)). Therefore, the degree of clustered nanocrystals is much higher in the experimental situation, in particular, if a high number of particles is deposited on top of the sensor surface.

This observation allows for different conclusions: a) A linear increase in the ΔTMR-response originates from dispersed particles which are sufficiently far away from other objects and, therefore, their magnetism is dominated by external fields prior to interparticle coupling. b) In the high concentration regime, dipolar coupling plays the major role for the dynamic processes and the equilibrium configuration of magnetic particles assembled in monolayers.

### 3.2 Transport properties

By embedding magnetic nanoparticles in non-magnetic matrices, they form the components of granular systems which reveal spin-dependent transport phenomena. Depending on the material of the interparticle matrix, different effects may occur: Conducting matrices result in giant magnetoresistance (GMR), the use of an insulating material in tunneling magnetoresistance effects. Ever since the discovery of the GMR-effect in granular Co/Cu-systems in 1992 by Xiao et al. [72] and Berkowitz et al. [73], numerous preparation methods have been introduced. Typically, granular materials are prepared by top-down methods such as co-sputtering or co-evaporation of matrix and precipitated materials as well as by metallurgic techniques [74–78].

A first bottom-up approach for the preparation of granular structures is based on the simultaneous deposition of particles, which are prefabricated in the gas phase, and the matrix material on a cold surface [79]. This approach has allowed for the avoidance of paramagnetic impurities within the matrix material and for the investigation of the dependence of the magnetoresistance effects on the particle size and volume ratios for different material systems [79–81]. Recently, Tan et al. showed that chemically synthesized, ligand stabilized nanoparticles can also be used for a bottom-up preparation of granular TMR systems [8–9]. An electrically isolating ligand shell acts as a tunneling barrier. TMR amplitudes of up to 3000% at low temperatures have been reported in such granular three-dimensional self-assembled supercrystals consisting of FeCo nanparticles (compare Figure 8(b)).

**Figure 14:**
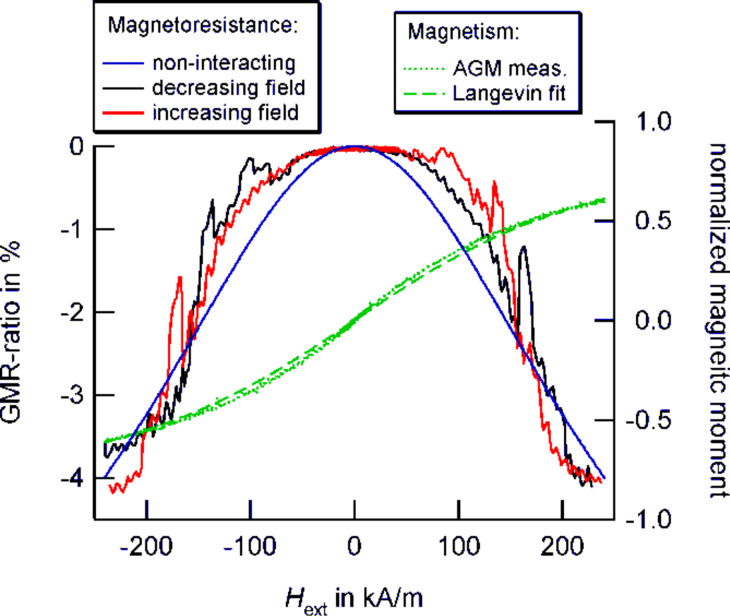
GMR response of a monolayer consisting of 8 nm Co particles covered by a thin Cu film. Measurements were taken at room temperature with a sample current of 1 mA and an in-plane external magnetic field. In comparison to the prediction of non-interacing particles, the experiments show additional features at field values of about ±176 kA/m, ±136 kA/m and ±88 kA/m.

In our work, we focus on the resulting transport properties of two-dimensional monolayers of Co nanocrystals embedded in a conducting matrix. Therefore, 8 nm Co particle assemblies have been created in a dropping procedure as described in Section 2. After the self-assembly process, the insulating ligand shells were removed by heating the particles for approximately 4 h at 400 °C in a reducing gas atmosphere. Subsequently, a thin Cu layer was deposited on top of the nanocrystals. The measurements were taken at room temperature via a four-point-measurement geometry; the results are shown in Figure 14: A GMR-amplitude of about 4% was observed with a bell shaped measurement characteristic.

In order to get a first qualitative understanding of the observed behavior, AGM measurements on the particles were carried out to determine the magnetic properties of the nanocrystals. As shown in Figure 14, the Co particles mainly exhibit a superparamagnetic behavior, their response to an external magnetic field follows the Langevin function. For non-interacting particles, the magnetization reversal may be employed to deduce the expected magnetoresistance characteristic in granular structures by

[13]
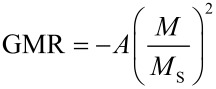


where *A* is the effect amplitude [78]. By comparison of the expected behavior according to Equation 13 and the experimental results (see Figure 14), additional features can be found in the measurements. Such features appear symmetrically for in- and decreasing external field strength and may be attributed to a dipolar coupling induced magnetic reversal of large coupled areas and, thus, the inner magnetic structure of the particle assemblies.

**Figure 15:**
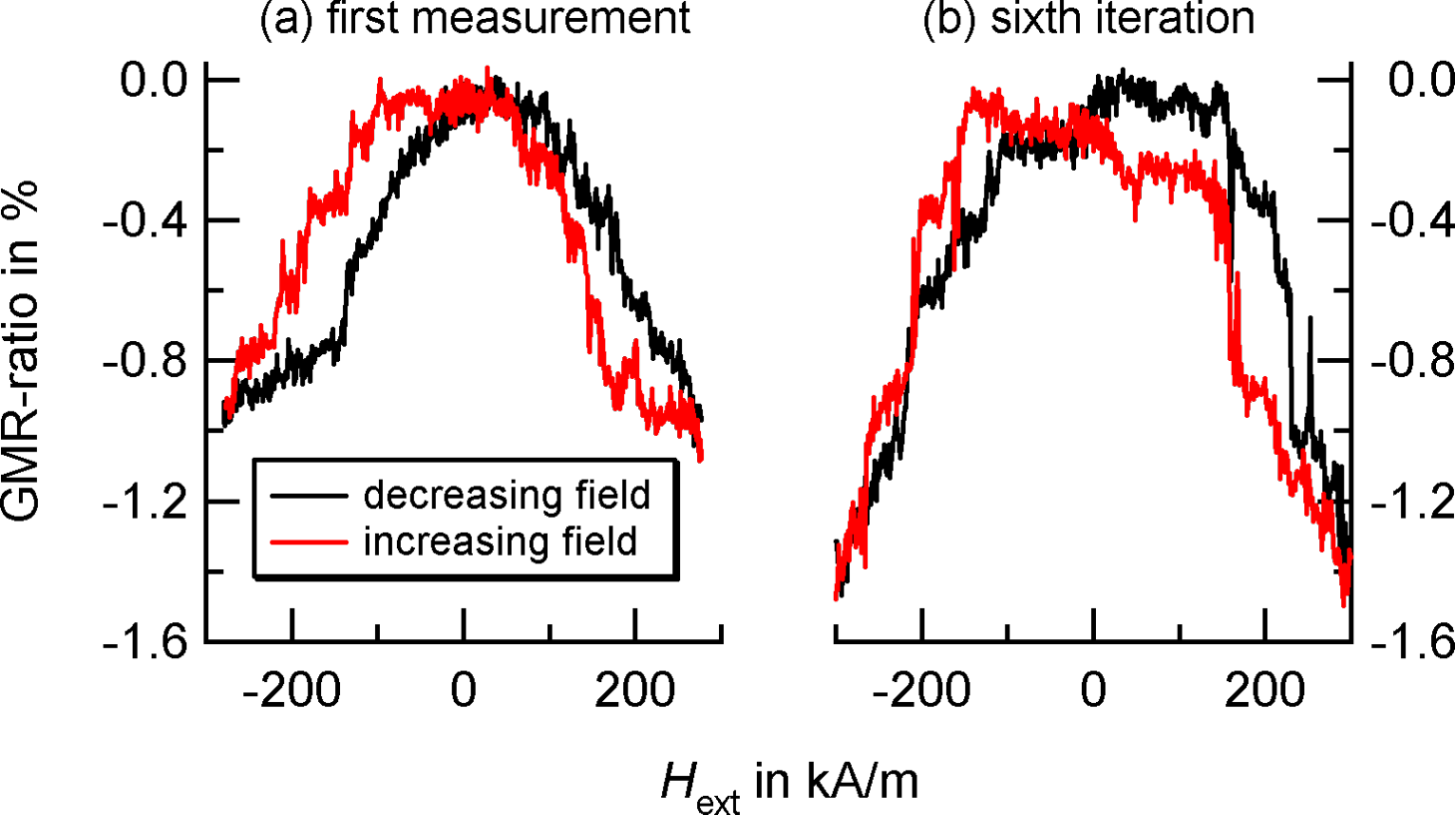
Magnetoresistance measurements at room temperature on a granular system consisting of Co nanoparticles with a mean diameter of ‹*D*› = (14.9 ± 0.4) nm covered with a 4 nm thick Cu-layer. The measurements have been taken from a series (a) at the very beginning and (b) after six runs.

As we will see in Section 4, the orientations of magnetic moments in such two-dimensional assemblies are correlated along domains with an antiparallel orientation similar to ferromagnetic materials. Consequently, the evolution of the magnetic configuration strongly depends on the history of the magnetic pattern and repeated measurements made under identical conditions may result in significantly deviating findings. An example obtained from a self-assembly of ferromagnetic Co nanocrystals (see Figure 10(e)) is shown in Figure 15: The first measurement (Figure 15(a)) resembles the behavior of non-interacting particles. On the microscopic level, the degree of correlation is very low and each magnetic moment exhibits a Langevin-like behavior. Due to the induced formation of domains, subsequent measurement increase the degree of dipolar coupling which entails a strong correlation between adjacent moments. The changes in the GMR-ratio occur step-wise, in particular, a broad plateau around *H*_ext_ = 0 may be reported. This observation corresponds to an antiparrallel arrangement of magnetic moments which maintains stability against external influences.

### 4. Particle based magnetoresistive sensors

**Figure 16:**
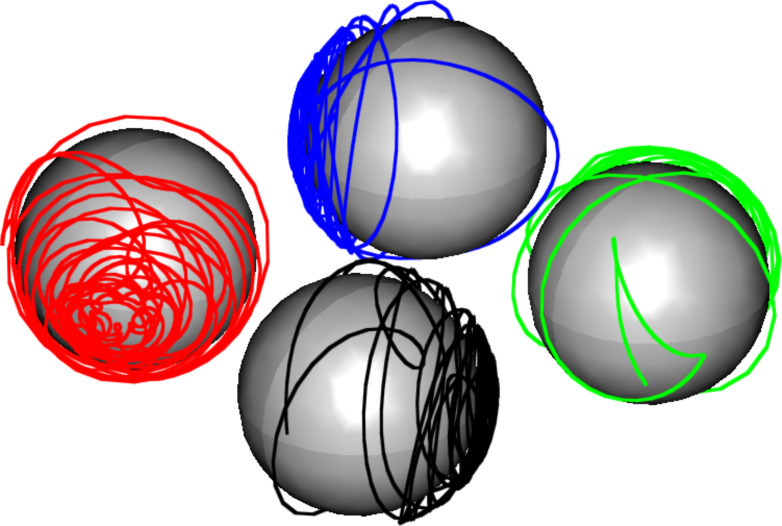
Magnetization evolution of four interacting magnetic dipoles arranged in the corners of a square with side length *a* = 15 nm. Dipole strengths are chosen equivalent to nanoparticles of radius *R* = 5 nm and *M*_S_ = 200 kA/m, the damping constant is set to α = 0.02. The stable equilibrium is reached after a timescale of 100 ns and is comparable to a vortex state which entails a very low stray field energy.

In a similar way as a small magnetoresistive sensor opens techniques for the design of a magnetic microscope in order to detect magnetic beads and particles and to evaluate spatial coordinates [82], an analogous approach should be possible for two-dimensional assemblies of magnetic nanoparticles. The principal idea in both strategies is very similar: An undisturbed reference configuration is exerted to some sort of perturbation which results in a variation of the magnetic configuration and, consequently, in a measurable resistance change. Since the measured signal depends on the magnetic field along the sensor area, it is possible to conclude the properties of the source. The key difference between the two approaches is a direct consequence of the governing Equation 12 for the evolution of discrete magnetic moments and the continuous equations for micromagnetic systems [83–84]: As shown in Figure 16, four discrete magnetic moments arranged along the corners of a square tend to align in a vortex-like state in order to minimize the total stray field energy of the system. Such behavior is not possible for ferromagnetic systems on the nanoscale. The interatomic exchange energy entails a strong confinement between neighboring spins which results in a strong magnetic stiffness on the mesoscale. Therefore, magnetic domains can only be found above a certain geometrical size scale; this is also the reason why the electrodes of the sensors discussed in Section 3.1 show no domain substructure. Due to such stiffness, elements are no longer sensitive to small field variations, which is one of the major challenges to overcome when downscaling magnetoresistive sensors below the micron range [85]. By employing assemblies of superparamagnetic particles, the confinement is broken in the most intuitive way - by spatial separation. Each particle forms its own magnetic domain, coupled to particles nearby via dipolar interactions [86]. This setup allows for localized switching of single magnetic moments and, therefore, forms a promising strategy for the design of increasingly smaller sensors. However, in order to guide future experiments and design new applications, a thorough analysis of the resulting properties is necessary. Therefore, in this final paragraph, we will study the response properties of these assemblies by solving the micromagnetic equations.

### 4.1 Equilibrium states and response functions

**Figure 17:**
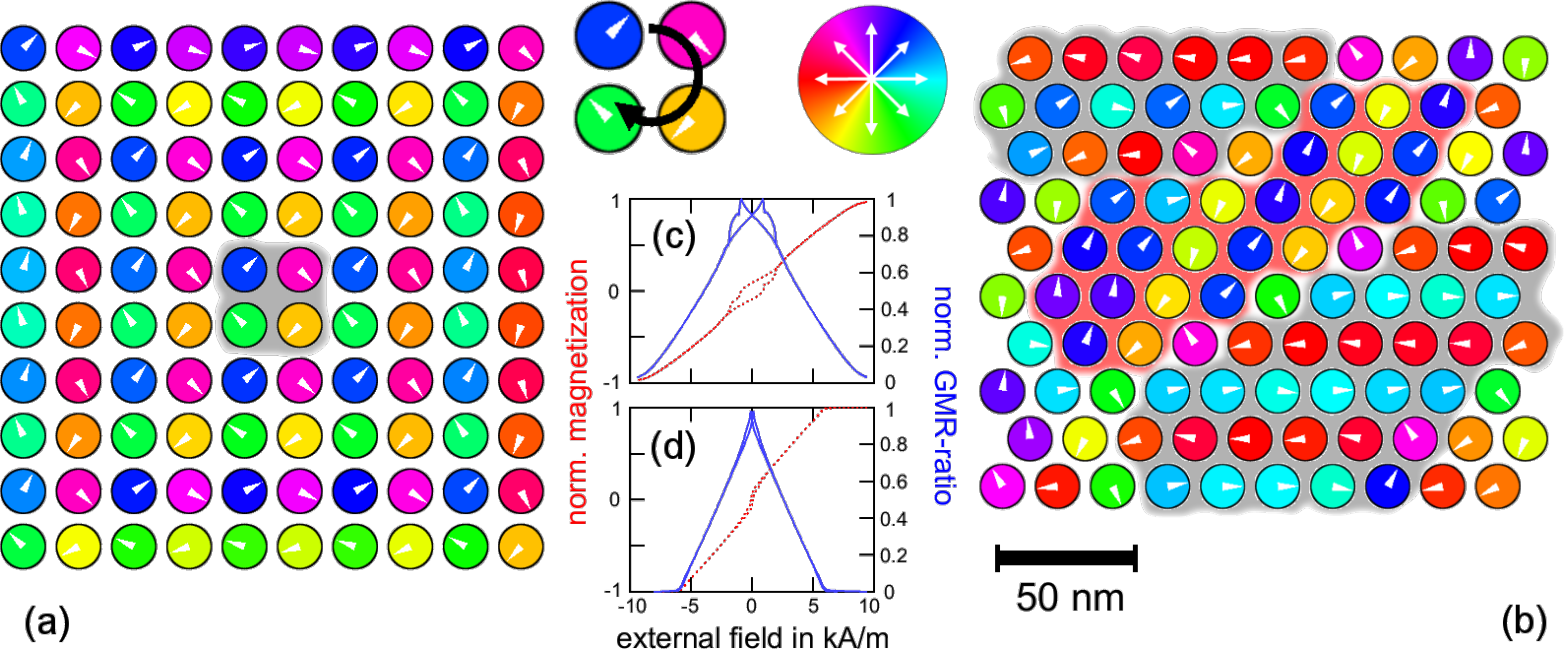
Equilibrium states of 10 × 10-particle arrays with cubic and hexagonal symmetry. Magnetic moments in cubic particle assemblies align in vortex-like 2 × 2-states while hexagonal symmetries entail domains of antiparallel ordering. The stability of the vortex states against external perturbations result in a hysteretic magnetization/GMR behavior, while hexagonal arrays have a linear behavior.

**Figure 18:**
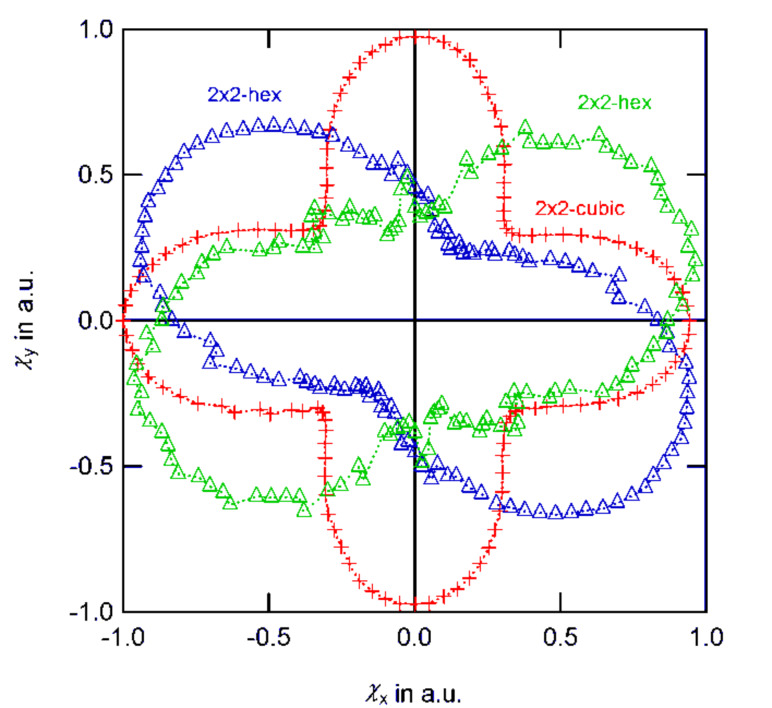
Direction dependent responses of different small particle assemblies to an external magnetic field. 2 × 2-grids align their magnetic moments in vortex-like states as shown in Figure 16. For cubic symmetry, the susceptibility is “degenerated” and independent of the vortex orientation. For hexagonal grids clock- and counter clock-wise orientation entail different responses.

As already demonstrated in the preliminary sections, individual magnetic moments are coupled to their neighbors via their dipolar stray fields. In contrast to the exchange coupling within a ferromagnetic material, such electromagnetic interaction entails an antiparallel correlation within the 10 × 10-particle array as shown in Figure 17 for the example of a cubic and a hexagonal grid: The out-of-plane components of the equilibrium moment distribution may be neglected. Therefore, the color code resembles the in-plane direction of the magnetic moment of each individual particle. The degree of local ordering varies between the two different grid types: A cubic symmetry decomposes into vortex-like substructures as shown in Figure 16. Close to the cluster edges, antiparallel moment loops are formed with the moment direction orthogonal to the boundary normal. Such elementary vortices are very stable against external influences which results in a hysteretic magnetization response as shown in Figure 17(c). Contrary, hexagonal assemblies show almost no hysteresis which is due to a different equilibrium state. Within the hexagonal lattice, magnetic domains are formed (Figure 17(b), highlighted areas) similar to the domain formation in ferromagnetic materials. However, the correlation leads to an antiparallel alignment where the magnetization direction follows lines of adjacent neighbors; the geometrical symmetry introduces a magnetic anisotropy. Consequently, the response of such setups to an external perturbation strongly depends on the direction of the applied magnetic field. Figure 18 shows the dependency of the susceptibility χ on the direction of an in-plane magnetic field for small particle assemblies. For cubic symmetry, the magnetically soft axes correspond to the grid vectors. Similar to the GMR measurements shown in Figure 14 where features occurred symmetrically for in- and decreasing field strengths, the response function χ is conserved and under a field rotation of 180°. For larger patterns, geometrical properties such as spatial configuration, the shape of the boundary or lattice distortions as well as the internal magnetic structure have a major impact and may result in various features.

The GMR ratio of such a magnetic pattern may be calculated according to V. Wiser as [87]

[14]
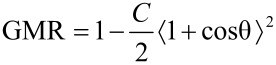


where the constant *C* is a measure for the spin dependence of electron scattering and θ the angle between adjacent magnetic moments. For the sake of simplicity, we will set *C* = 1 in the following. Due to the antiparallel alignment and domain formation, a high degree of magnetic disorder is obtained if there is no external magnetic field applied. Therefore, the equilibrium state entails a high resistance according to Equation 14. Under the influence of a magnetic field, a configuration of increasing order and decreasing resistance is obtained as we already learnt from the above transport measurements.

These observations form the conceptional basis of a granular giant magnetoresistance (gGMR) sensor. The stray field of a magnetic bead outside the assembly results in the partial alignment of the magnetic moment vectors; the degree of alignment depends on the position and the material parameters of the external object.

### 4.2 Spatial resolution properties

In order to analyze the capability of a two-dimensional particle assembly as a gGMR sensor, we consider a similar hexagonal particle patch as shown in Figure 17. The equilibrium state of the magnetization is calculated by solving Equation 13 under the influence of an additional probe particle *P* modeled by Equation 10. We denote the centre coordinates of *P* by **r**_P_, the radius by *R*_P_ and the magnetization by *M*_P_. For the evaluation of the position influence, the particle centre is placed along the nodes of a discrete grid, with grid nodes at

*x*_P_ = {−200 nm + *i* • 8 nm, *i* = 0, … , 50}

*y*_P_ = {−200 nm + *j* • 8 nm, *j* = 0, … , 50}

*z*_p_ = 100 nm

For a first analysis of the response properties, we make two simplifying assumptions: a) we can manipulate the magnetism of the probe particle without influencing the particle assembly itself and b) we can directly deposit the particle at a certain node point. The first assumption is legitimate if a magnetization perpendicular to the sensor plane is imposed. From simulations, we learn that the susceptibility χ_z_ is very small; an external magnetic field employed to bring particles into saturation only has a small effect on the magnetization distribution within the particle assembly due to the strong in-plane confinement. For in-plane components, this is no longer true. Therefore, these simulations may only be taken as a first estimation on the expected behavior. Further, the second assumption is not valid in the experimental situation. The iterative measurements on identical systems have revealed a strong dependency on the history of the magnetic state (Figure 15). We will not use this simplification in Section 4.3 in order to estimate the impact of hysteresis within these setups.

**Figure 19:**
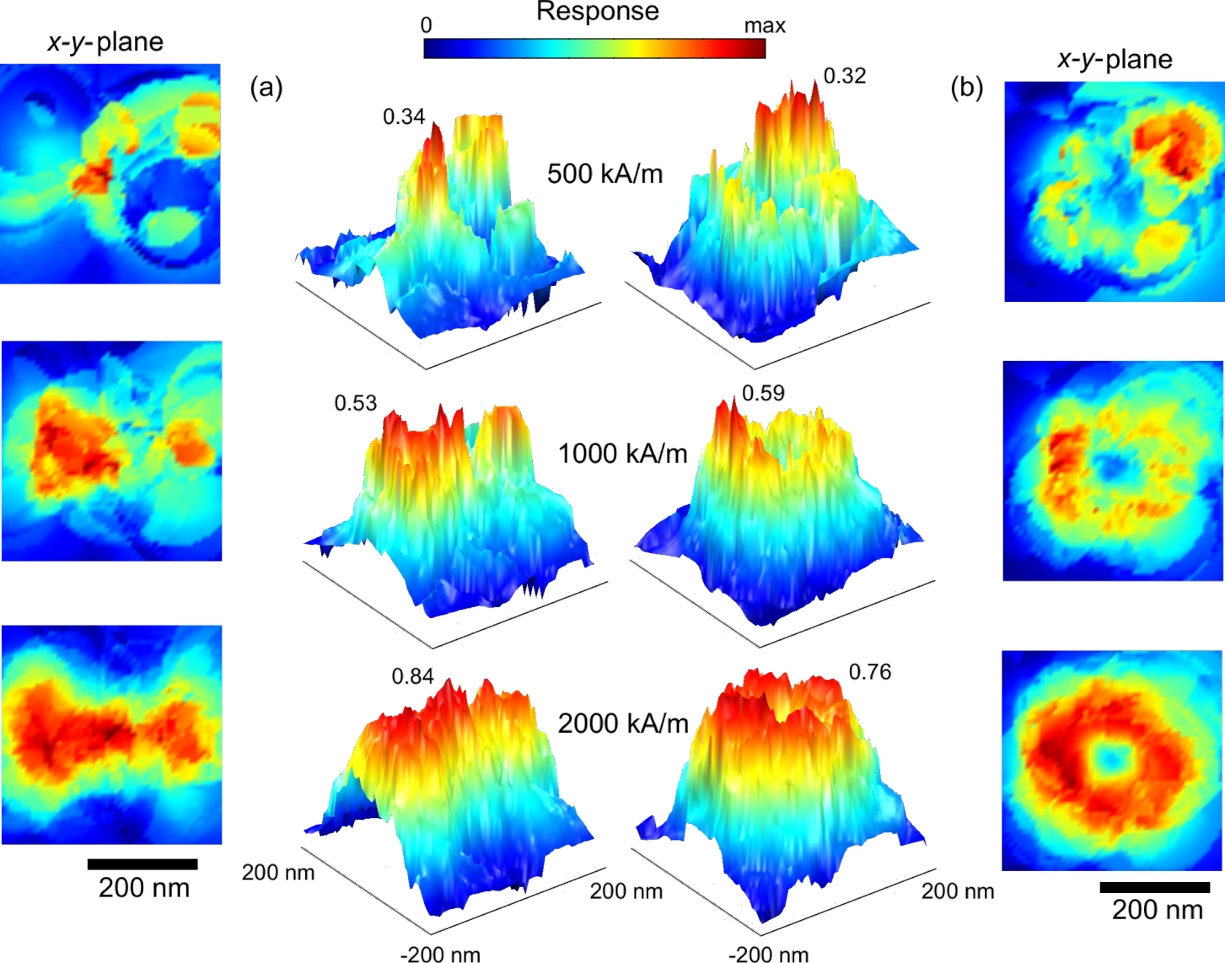
Response maps of a 10 × 10-hexagonal gGMR sensor for a probe particle with *R*_p_ = 50 nm and *M*_p_ = 500 (top), 1000 (middle) and 2000 kA/m (bottom); (a) shows the response for **m**_p_ || 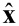 and (b) the results for **m**_p_ || 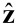.

By solving Equation 12 for the probe particle at a certain grid point the respective GMR value can be calculated from the solution according to Equation 14. The results are shown in Figure 19 for a 10 × 10-hexagonal gGMR sensor consisting of *R* = 8 nm particles of a magnetization *M* = 1000 kA/m; values in between the discrete nodes are obtained by linear interpolation. The probe particle is chosen with radius *R*_p_ = 50 nm and a magnetization half, identical and double to sensor components. By such variation of the perturbation strength, we may identify two different characteristic behaviors/measuring modes of the granular sensor:

For a magnetic particle with a sufficiently strong magnetic moment (Figure 19, bottom), the response surface resembles the crosscut of the particle stray field. The influence of the particle is strong enough to overcome the interparticle dipolar coupling within the sensor which results in global switching of the entire plane and, consequently, in very high response ratios. For very low particle strengths (equivalent to particles very far away from the sensor), the coupling within the particle plane remains dominant. The imprint of the stray field may still be identified but in common with MR sensors, it will fall below the noise value of the device. However, here the major advantage is revealed. Along the sensor, regions of high response sensitivity are present which enable the detection of much smaller magnetic fields.

Further, the results also reveal that the reduced stiffness is bought at a certain cost: In contrast to similar response maps for TMR sensors [65], the gGMR maps are not smooth. The discrete particle assembly entails an inherent “deterministic” noise contribution which was also present in the experimental realization (compare Figure 14 and Figure 15). These additional features originate from localized switching events and the discrete spatial structure of the gGMR sensor.

### 4.3 Hysteretic particle monitoring

A major advantage of the gGMR sensor lies in the strong capability of the magnetization distribution to perform local switching. Therefore, the assumption of directly placing the particle at a certain node point allows for a first qualitative understanding of the expected results but will not resemble the quantitative situation particularly well. In order to obtain a first estimation on the importance of hysteretic behavior, we assume the probe particle to travel from one side of the sensor the other one along

*x*_P_ = {−250 nm + *i* • 2.5 nm, *i* = 0, … , 200}

*y*_P_ = 0 nm

*z*_p_ = 100 nm

**Figure 20:**
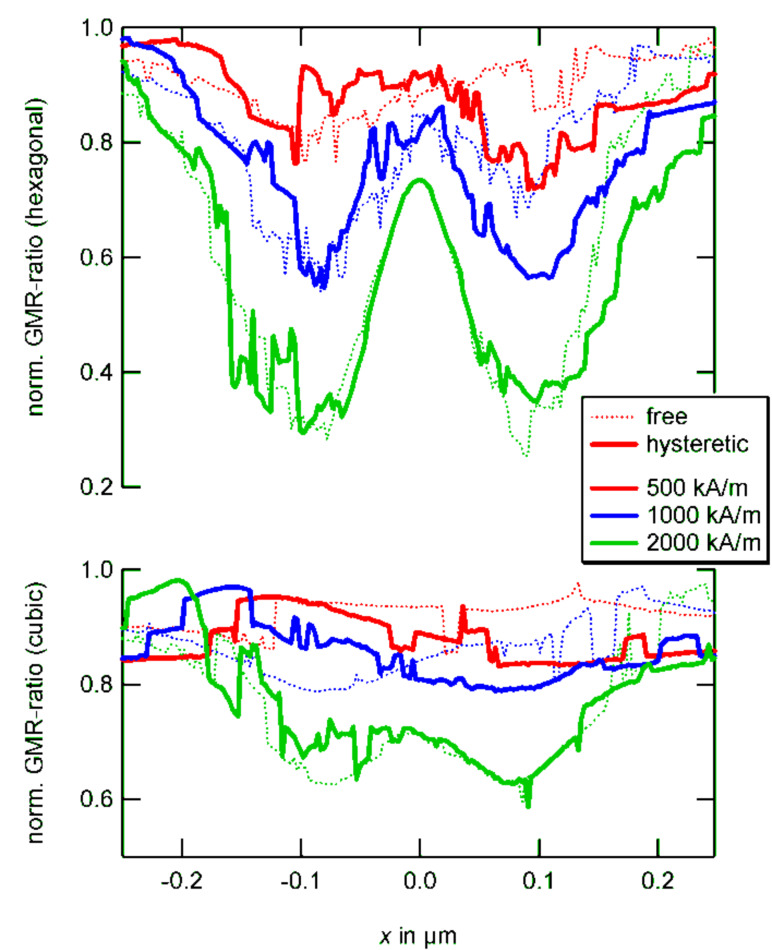
Comparison between free (dotted) and hysteretic (line) sensor behavior for cubic and hexagonal symmetries. Due to stable vortex-like states, the overall effect is smaller for the cubic assemblies. For high particle moments, hysteretic and non-hysteric responses show only small deviations.

The resulting set of equations is solved in same way as before. The results are shown in Figure 20. Intuitively, it could be expected that memory effects gain importance, the higher the magnetic moment of the source to be detected. In general, the numerical results show that this assumption is not true (compare Figure 20, 2000 kA/m). Instead, above a critical threshold, interparticle coupling is diminished and the hysteretic behavior resembles the non-hysteretic situation. Independent of the particle position, a switching along large areas of the sensor takes place.

A different observation can be made for lesser source impacts: If the particle migrates along a path where no areas of enhanced sensitivity are crossed, only small deviations in the hysteretic and non-hysteretic response can be found. However, by passing a hot spot in the gGMR map, a permanent change in the magnetic configuration is entailed and consequently, a discontinuous jump in the gGMR response is the result (compare Figure 20, cubic, 500 kA/m; 1000 kA/m); the measured value evolves from there on along a different branch.

## Conclusion

We have shown how magnetic particles synthesized by bottom-up methods may form the components for granular GMR sensors. Due to their narrow size distribution, various preparation methods allow for the manufacturing of long scale, highly symmetric monolayers of magnetic nanocrystals. Along these assemblies, dipolar coupling is the dominating driving force for their magnetic properties and the resulting behavior of the ensemble. Embedded in a non-magnetic matrix, a spin-dependent transport occurs which forms the conceptional basis of the gGMR sensor. Due to spatial separation of individual nanoparticles and the entailed missing of exchange coupling, the magnetic stiffness of ferromagnetic thin film systems is overcome and areas of enhanced sensitivity are introduced along the sensor surface. A thorough analysis of these hot spots and the different possible switching states will prove key in the future development of the gGMR approach to the design of nanoscale detection devices.
